# The Treatment of Gonococcal Infections

**Published:** 1919-11-22

**Authors:** 


### THE TREATMENT OF GONOCOCCAL INFECTIONS.

### A Review of Modern Developments.

In spite of the recent controversies which raged round the advisability of prophylaxis in the case of this infection, and whatever excellent results as to reduced incidence, etc., we may expect from such publicity, the fact remains that the practitioner will probably now, and in future, continue to meet many more gonococcal cases than either he or the country would wish, and in view of the unmistakable fact that more than a few medical men do not realise the precise importance and exact value of certain recent advances in treatment, we need offer no excuse for summarising a few of the outstanding points in this connection.

### Film Preparations.

The Medical Research Committee, in a special report on the Laboratory Diagnosis of Gonococcal Infections, have issued the following recommendations, and simple though they are, it is not certain that their meaning is fully and generally utilised in practice. The diagnosis of gonococci in film preparations cannot be based on the presence or absence of intracellular diplococci, even of characteristic shape, for two reasons, (1) the gonococci may be *extracellular* both in acute and chronic gonorrhœa; (2) other organisms in the genito-urinary passages may be intracellular. The only reliable method is the simple Gram stain; the gonococcus is Gram-negative—and most organisms with which it may be confused Gram-positive. Even the original Gram method itself is liable to many errors such as (1) If the film be too thick, decolorisation may be incomplete; (2) If the film be unduly heated very doubtful results may be given; (3) If aniline-water-gentian-violet is not fresh it does *not* give a differential stain; (4) If the alcohol is not "absolute" excessive decolorisation may occur, and so on with a host of limitations due chiefly to the use of degenerated or evaporated stock solutions, a circumstance more than liable to arise in the practitioner's home laboratory.

A method strongly advocated, and by our own experience most satisfactory in practice, is that of Jensen. The gentian-violet is replaced by a half per cent. aqueous solution of methylviolet, the iodine is increased to iodine one part, potassium iodide two parts, distilled water 100 parts, and neutral red is used as a counter-stain, by which means much cleaner staining is obtained in the hands of the inexpert. Of course, the acute infection is readily diagnosed under most circumstances, but in chronic, latent, and recurrent cases, there is no little difficulty, and it must be realised that among the diagnostic limitations of Gram methods fall, (1) Gram-positive organisms may lose their character when digested by leucocytes; (2) They give no differentiation between gonococci, other Gram-negative diplococci, non-pathogenic, which are in practice quite frequently encountered under similar conditions. Thus recourse must often be had to other methods, which although most frequently mentioned, are almost as often misunderstood.

### Diagnostic Reactions.

For example, there is the attempted production of a focal reaction in doubtful cases by deliberate inoculation of a high dose of gonococcal vaccine. As a means of detecting latent gonorrhœa of fairly recent origin, this procedure is undoubtedly dangerous. Apart from the liability to relight the trouble far in excess of diagnostic needs, there is a definite possibility of starting a latent iritis or salpingitis. The only conditions under which we have seen a really efficient application is in the instance of the later stages of the latent type—namely, old gonorrhœal arthritis, and this in instances where the above-mentioned distributions have with certainty never previously appeared. Small vaccine doses will definitely produce transient pain reactions, which are most valuable in deciding at least the origin of the trouble.

The complement fixation tests in gonorrhœa, the tests comparable to the famous Wassermann reaction, must be described as valuable only when expert laboratory service is at hand, and as to their relative value one may quote the official decision on the subject. The greatest utility comes obviously in the instances of metastatic infection, where direct demonstration of the organism is well-nigh impossible. Even then certain definite limitations must be realised. (1) Acute infection gives no reaction until three or four *weeks* after the onset; (2) Chronic cases may give negative results when the disease is closely localised; (3) In women little or no reaction is obtained until the disease has spread to the uterus; (4) It is useless in cases using vaccines, as the blood in such cases may continue for four months to give the reaction; (5) Experiments in animal immunity have proved that positive reaction may be obtained several weeks *after* cure is complete.

### General Treatment.

In the ordinary simple methods of local treatment little change has been made by the last few years' observations. One or two points may be mentioned, not for their novelty but rather for their essential importance. Alkaline drinks should be avoided, since an acid urine is a safeguard against a secondary cystitis. Irrigations may with advantage be given as hot as can be borne, and later, when the urine is free from pus, a provocative injection of silver nitrate is advised. As an adjunct to the fully-established urethroscopic methods, there are now on the market graduated mechanical dilators with a combined suction apparatus—such as the Mills' instrument—which are simple and most useful in the hands of the unspecialised worker. Remember that posterior urethritis is not unfrequently due to too vigorous local treatment, and that should epididymitis occur, it is advisable at

**The Treatment of Gonococcal Infection**—(*continued*).

once to stop urethral treatment. A point in prostatic massage treatment is that, in order to lessen the danger of epididymitis as a result of this procedure, it is advisable to irrigate the urethra and fill the bladder before commencing massage.

In the *Practitioner* of May 1918, McDonagh gave some remarkable results of intramuscular injections of colloidal manganese and antimony. The stay in hospital was undoubtedly reduced by this treatment consisting of five injections of manganese given at intervals of two or three days in 1cc. to 2.5cc. doses. If the discharge persisted, colloidal iodine of ʒiij thrice daily, was given, and also colloidal antimony in the eighteenth and twenty-second days, in doses of 1.5cc. of a 0.04 per cent. emulsion. The manganese is said to act mainly on the common complicating staphylococcus infection, and to be strictly an adjunct to general local treatment. Certainly it was remarkably successful in several extensive complications, and the work has been of considerable value, even if somewhat unsuitable for general adoption in practice.

### The Use of Vaccines.

There is little doubt that in acute gonococcal conditions ordinary vaccines have been of but small avail, and have long ago been so severely criticised that further mention is here unnecessary. We can, however, testify from experience that in cases of the later manifestations when the action and the reaction to the infection is chronic and relatively balanced, most successful results may be obtained by ordinary, or better, by simple "sensitised" vaccines, which latter can be pushed without causing undue local or general reactions. The latter are undoubtedly less toxic, and seem to have at least some value in the typical acute attack, while in the typical arthritis cases we have seen some truly rapid and excellent benefits result. Of the more recent products the so-called "detoxicated" vaccine, in which the organisms concerned undergo such drastic dissolving treatment in the process of preparation, that it is hard to believe the final inoculation can have any properties capable of provoking anti-bodies specific to the original gonococcus, we must, at this early stage in their history, reserve our final judgment. This type of vaccine is already being widely used, and still more widely advertised; but scientifically there is yet more observational work to be done on the true effects before their many critics will be satisfied. Doubtless their success is considerable, but many unbiased opinions are heard to the effect that the simple "sensitised" products commonly made in all laboratories are as efficient, if not more so.

